# History and structures of telecommunication in pathology, focusing on open access platforms

**DOI:** 10.1186/1746-1596-6-110

**Published:** 2011-11-07

**Authors:** Klaus Kayser, Stephan Borkenfeld, Amina Djenouni, Gian Kayser

**Affiliations:** 1 Charite, University of Berlin, Berlin, Germany; 2 IAT (International Academy of Pathology), Heidelberg, Germany; 3 Amina Djenouni, Batna, Algeria; 4 Institute of Pathology, University of Freiburg, Freiburg, Germany

**Keywords:** Telepathology, telemedicine, virtual slide, open access forum, MECES

## Abstract

**Background:**

Telecommunication has matured to a broadly applied tool in diagnostic pathology.

**Technology and Systems:**

Contemporary with the development of fast electronic communication lines (Integrated digital network services (ISDN), broad band connections, and fibre optics, as well as the digital imaging technology (digital camera), telecommunication in tissue - based diagnosis (telepathology) has matured. Open access (internet) and server - based communication have induced the development of specific medical information platforms, such as iPATH, UICC-TPCC (telepathology consultation centre of the Union International against Cancer), or the Armed Forces Institute of Pathology (AFIP) teleconsultation system. They have been closed, and are subject to be replaced by specific open access forums (Medical Electronic Expert Communication System (MECES) with embedded virtual slide (VS) technology). MECES uses php language, data base driven mySqL architecture, X/L-AMPP infrastructure, and browser friendly W3C conform standards.

**Experiences:**

The server - based medical communication systems (AFIP, iPATH, UICC-TPCC) have been reported to be a useful and easy to handle tool for expert consultation. Correct sampling and evaluation of transmitted still images by experts reported revealed no or only minor differences to the original images and good practice of the involved experts. β tests with the new generation medical expert consultation systems (MECES) revealed superior results in terms of performance, still image viewing, and system handling, especially as this is closely related to the use of so - called social forums (facebook, youtube, etc.).

**Benefits and Expectations:**

In addition to the acknowledged advantages of the former established systems (assistance of pathologists working in developing countries, diagnosis confirmation, international information exchange, etc.), the new generation offers additional benefits such as acoustic information transfer, assistance in image screening, VS technology, and teaching in diagnostic sampling, judgement, and verification.

## Introduction

Obviously, medical diagnoses and treatment depend upon human senses, especially visual and acoustic information. Visual information is the most frequently used "objective" diagnostic source whereas acoustic information is mainly characterized by the patient's senses and their linguistic terms [1,2]. These diagnostic information sources can be distinguished in four main components that include environmental data (home care, accidents, etc.), distinct patient's data (history, physical examination, behaviour, complaints), functional data (related to organ function, i.e., electrocardiogram, blood pressure, etc.), and structural data (computed tomography (CT), microscopic images etc.). A multifunctional telemedicine system should serve for all these different components, if possible [3-10].

Environmental data require wireless access, especially in emergency cases such as car accidents with immediate therapeutic interventions (for example advice to/exclusion of neurosurgery in a severe car accident).

Distinct patient's data might ask for live multimedia transfer (gerontology, care units for elderly), functional data might request the transmission of short term videos (acute heart infarction, etc.), and structural data the transmission of high resolution images [5,6,10-15].

In contrast to previous systems recently available forums should serve for all different demands. In addition, they might be adjusted to a panel of distinct aims. These include performance of primary diagnosis at potentially multiple places separated from tissue examination, processing and image acquisition from glass slides (frozen section service), confirmation, refinement of primary diagnoses (secondary diagnoses and diagnostic quality assurance by expert consultation), teaching and education of students and young colleagues (e-learning, education, and training), and on-line (live) teleconferencing [15-17].

Although some of the "older" systems (iPATH) claim to be applicable for all these different purposes they are most frequently used for expert consultation, and rarely for other aims such as education [14,18-21].

In this article we want to describe and analyze the development of electronic information transfer, and the properties of recently released applicable tools such as the open access forum MECES, which are constructed in a manner similar to so-called social forums (facebook, youtube, etc.).

## History and Mile Stones of Telemedicine/Telepathology

**Figure 1:**
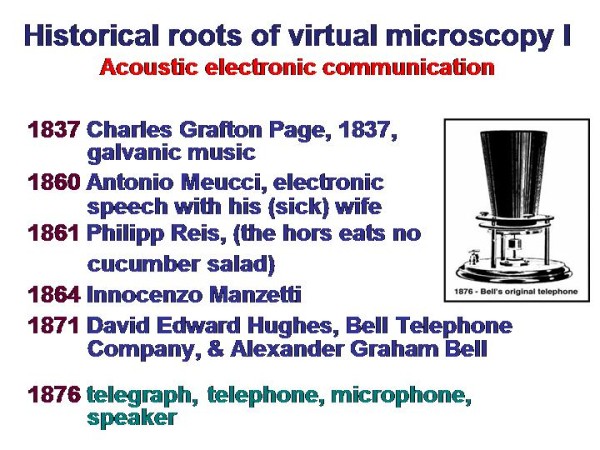
**Historical roots of virtual microscopy related to acoustic electronic communication**. The virtual slide for this article can be found here: http://www.diagnosticpathology.diagnomx.eu/vs/1617760077623140/1.

**Figure 2:**
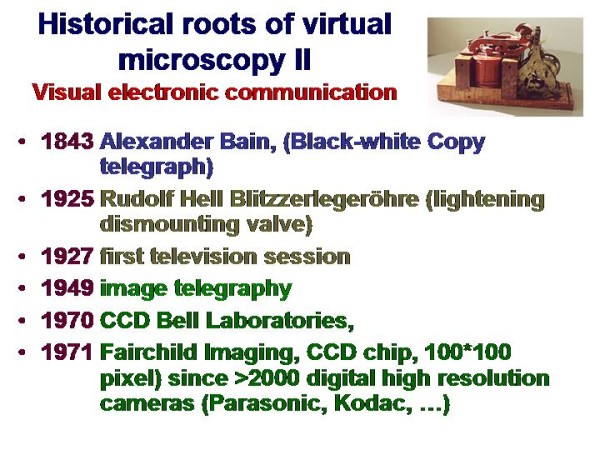
**Historical roots of virtual microscopy related to visual electronic communication**. The virtual slide for this article can be found here: http://www.diagnosticpathology.diagnomx.eu/vs/1617760077623140/2.

**Figure 3:**
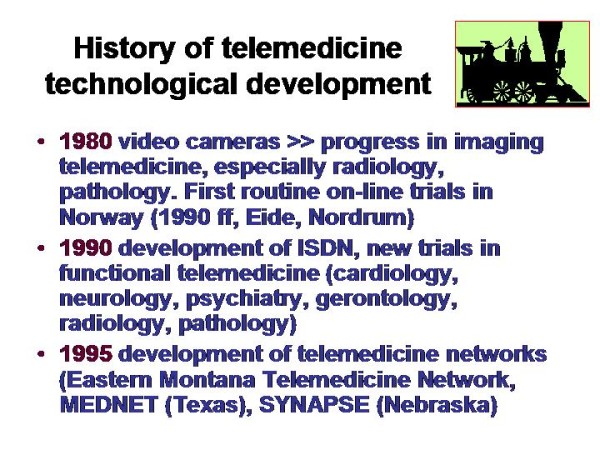
**History of telemedicine related to the technological development**. The virtual slide for this article can be found here: http://www.diagnosticpathology.diagnomx.eu/vs/1617760077623140/3.

**Figure 4:**
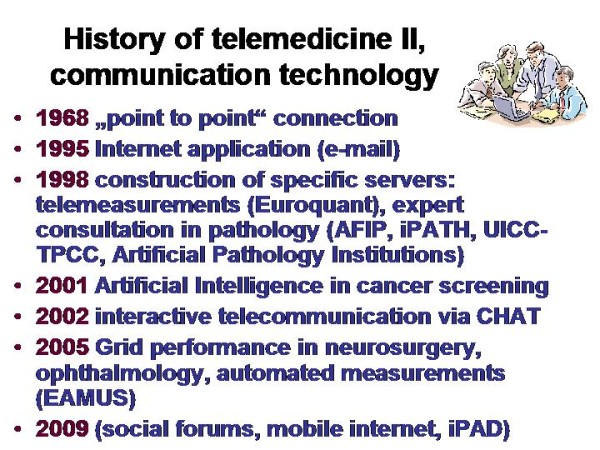
**History of telemedicine related to the communication technology**. The virtual slide for this article can be found here: http://www.diagnosticpathology.diagnomx.eu/vs/1617760077623140/4.

Telemedicine is an electronic information transfer of images ands sounds. It requires, in general, low physical energy and fast connection lines (and embedded systems such as servers). Its roots range back for more than one hundred years, as demonstrated in Figure 1 and 2. The first trials in sending and receiving acoustic signals (speech) have been undertaken by Charles Grafton Page in 1837, who has been able to transfer "galvanic music"; those of transferring visual data (images) nearly at the same time by Alexander Bain, who constructed a black - white telegraph. A detailed description of the amazing history can be found in Beauchamp, and in Huurdeman [1,2]. Thus, the principle roots of telemedicine range back for more than 150 years. However, technical matured systems are commercially available only since the development of fast connection lines and digital cameras in the 1990s, as demonstrated in Figure 3 and 4.

**Figure 5:**
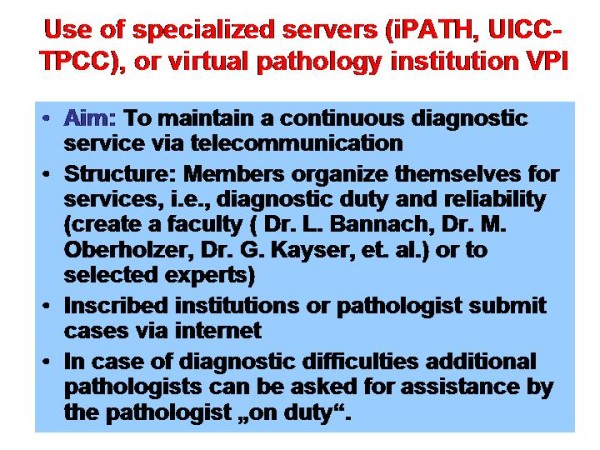
**Survey of organization of a virtual pathology institution (VPI)**. The virtual slide for this article can be found here: http://www.diagnosticpathology.diagnomx.eu/vs/1617760077623140/5.

**Figure 6:**
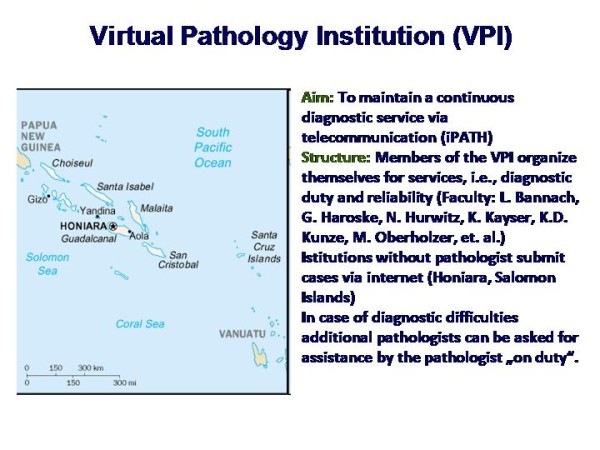
**Data of the former VPI Salomon Islands**. The virtual slide for this article can be found here: http://www.diagnosticpathology.diagnomx.eu/vs/1617760077623140/6.

The history of telepathology and telemedicine specific applications is depicted in Figure 5 and 6. Of major significance are the establishment of fast telephone lines starting with ISDN, the mass production of digital cameras, and the standardization of the open access network (internet) [4-6,8,10,11,14,19,22-26]. Based upon these three components several telemedicine systems have been constructed at the end of last century [7,8]. The best known and most frequently used are the AFIP system in Bethesda, USA, the iPATH, developed by Brauchli and Oberholzer at the Institute of Pathology, University of Basels, Switzerland, and the UICC-TPCC developed by Dietel, Hufnagl, and Schrader at the Institute of Pathology, Charite, Berlin [3,12,15,20,21,27-29]. These systems have been constructed to primarily serve for expert consultation; they can, in addition, be used for teaching and education too [24,30]. They differ in certain details such as responsibility of the expert in relation to the client, selection of experts by the client, internal flexibility, although all of them follow the same principle: to use the internet for (mainly) visual communication with clearly separated duties of the client and of the expert [7,8,13,23,28].

The iPATH, the most flexible system has been most frequently used, and more than 8,000 consultations have been reported [3,7,13,18].

In this regards, it is of specific interest that the iPATH has been chosen for implementation of a Virtual Pathology Institute (VPI). This unique pathology institute was internally organized similar to a conventional institute of pathology, i.e., there were colleagues on duty who served for the diagnostics in a distinct period (week), others remained in the "background" for additional assistance, if needed, or to replace the colleague on duty if he/she was unforeseen absent (sick).

This construct could maintain all diagnostic pathology services for the Salomon Islands (see Figure 5 and 6 for several years. Trained technicians handled the surgical specimens, provided the glass slides, and acquired the images which were submitted via the iPATH system to the colleague on duty. These colleagues were working in different European countries (Switzerland, Germany, Italy). The diagnoses were electronically transferred to the surgeon working in Honaria, Salomon Island. The glass slides were sent to colleagues working in Australia for confirmation/refinement of the diagnosis, which usually took two months all in all; the diagnoses of the VPI were released within 24 hours in general [7,13,31].

At present, the mentioned three telemedicine systems, namely AFIP, iPATH, and UICC-TPCC have been closed, or are no longer in a continuous maintenance: Unfortunately, the AFIP has been closed due to financial reasons, its teleconsultation platform is no longer available, and has partly been replaced by Telepathology Consultants http://telepathology.com. The UICC-TPCC has been completely closed due to financial reasons too, whereas the iPATH server due to internal reasons has been transferred to a new server hosted by Basis Data, a private company. In addition, it has been (partly) replaced by the new system Campus Medicus http://campusmedicus.net.

## Features of new solutions

The innovative features of the former iPATH telemedicine system include a) open software, b) flexible internal organisation in terms of group formation, expert selection, inclusion of search functions and data banks. Some new features have been added in its follower Basis Data which include access to libraries, discussion of case - independent issues such as conference announcing or chat, and videoconferencing.

**Figure 7:**
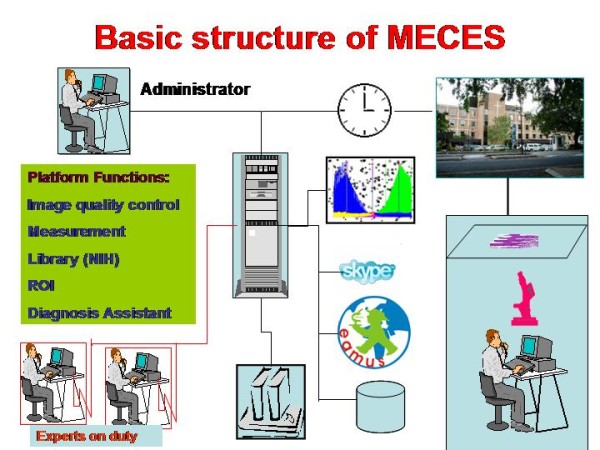
**Basic structure of MECES (Medical Electronic Communication Expert System)**. The virtual slide for this article can be found here: http://www.diagnosticpathology.diagnomx.eu/vs/1617760077623140/7.

**Figure 8:**
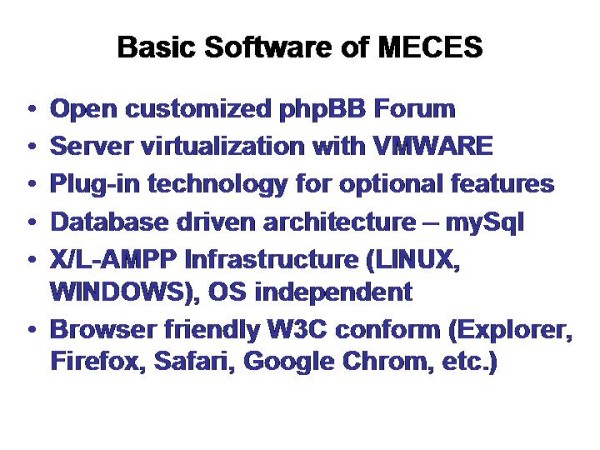
**Basic software of MECES**. The virtual slide for this article can be found here: http://www.diagnosticpathology.diagnomx.eu/vs/1617760077623140/8.

**Figure 9:**
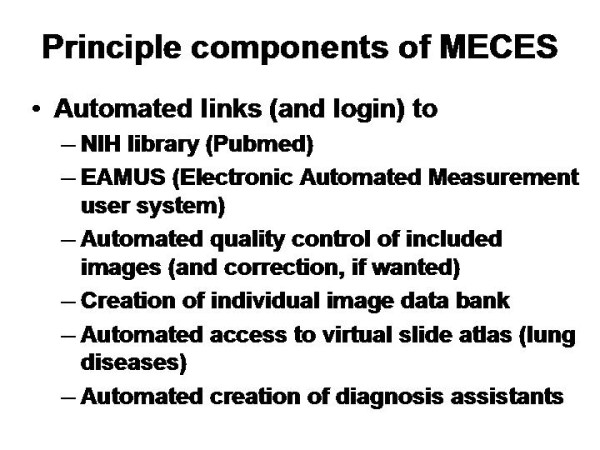
**Principle components of MECES**. The virtual slide for this article can be found here: http://www.diagnosticpathology.diagnomx.eu/vs/1617760077623140/9.

A new development of a telemedicine/telepathology system should meet several additional features [7,9,15,16], Based upon the practical experiences explored with the iPATH, UICC-TPCC, and the AFIP system, the items are displayed in its basic scheme Figure 7. The technical and content related details which would be of significant practical value if implemented in a forum derived medical expert consultation and education system (MECES) are listed in Figure 8 and 9:

Figure 8 displays the basic software of such a system, exemplarily demonstrated for the MECES system. It is constructed as an open customized phpBB forum that can be accessed by W3C conform browsers such as Explorer, Firefox, Safari, Google Chrom etc. The server is virtualized by VMWARE. The X/L-AMPP infrastructure permits an OS independent operation under WINDOWS or LINUX.

Figure 9 displays the principle components of such a (MECES) system: The basics of a forum (submit and reply) are associated to the medical partners (client and expert). Both of them are permitted to attach (image) files and write comments in a structured manner (i.e. patient's data, preliminary/final diagnosis, etc.). In addition, specific links are added that permit the use of external information sources and of quality evaluation of the submitted/included issues. These include a) an automated access to the National Institute of Health (NIH) library (pubMed). Herein the search term is automatically taken from the diagnosis field. This feature ensures the access to the latest scientific publications of the task under discussion.

b) Specific attention has been given to the included still image viewer: It is provided with an interactive navigation and magnification module allowing a microscope - like viewing of the attached still images. Commercially available VS images can be included and viewed too using the viewers of the specific companies, such as Leica http://www.leica-microsystem.com or 3DHistech http://www.3dhistech.com.

In preparation are a) an annotation module of still images, b) an image quality evaluation system for potential automated quantitative measurements, c) automated access to the Electronic Automated Measurement User System (EAMUS™), d) automated access to virtual slide atlases (in preparation is a VS atlas of lung and other diseases), and e) a link and transfer of images to a client specific diagnosis assistant data bank [16,32-34].

The multimedia approaches include the implementation of a separate acoustic information transfer (Skype) as well as teleconferencing.

All images and clinical data can be standardized using the Transmission Control Protocol/Internet Protocol (TCP/IP), i.e., by the DICOM - 3 (Digital Imaging and Communication in Medicine) standard [35-37]. A Picture Archiving and Communication Standard (PACS) is still under development as this standard has not definitively set up for application in surgical pathology (gross and microscope images) [38-41].

The basic use of the MECES system has been taken from the experiences in working with the iPATH or UICC-TPCC: All users have to register, and their access to the system has to be confirmed by an administrator. Clients and experts are automatically informed about new messages by email notification (or other communication pathways such as SMS, if wanted). The internal organization can easily be adjusted to the requirements of a VPI as described above.

## Experiences and Perspectives

The experiences of the described forum are in its β-phase. The potential users are already quite familiar with the social forums such as facebook or youtube, and seem to enjoy the MECES system as its performance is closely related to the mentioned social forums. The handling of enclosed images is superior to that reported from the already existing medical expert consultation systems.

The open access and source strategy as well as the chosen phpBB software, the database driven mySql architecture, and the X/L-AMPP infrastructure allow browser independent world wide access, in contrast to those programs that use HTML or other languages (Campus medicus) for example [3,13,19]. The perspectives of such a modular and open communication system in medicine are, to our opinion, two fold:

a) There is no doubt that the scientific and medical gap between developing and developed countries is increasing. The latest tissue based diagnostic procedures, namely the so called predictive diagnosis are expensive and require highly specialized pathology institutes [42-45]. The described forum derived systems such as MECES can assist to bridge the gap. They can be used to optimize information transfer between institutions working in developing and developed countries, to include external experts for diagnostic and therapeutic issues, and to steer the mandatory communication and potential molecular biology tissue preparation. In addition, after, or even contemporary with the analysis of still images, glass slides could be sent for VS analysis to a VS image acquisition centre that handles the necessary logistics, and provides the forum with the underlying VS.

b) The algorithms of expert consultation are, in general, close to those of education and teaching [8,23,40]. Therefore, it is reasonable to use medical expert consultation forums for education too. Education in tissue based diagnosis includes two different aims, namely the recognition of tissue alterations (sampling), and the association of the recognized changes with the underlying disease (diagnosis) [7,34]. Both items are different investigations, are, however, usually not taught separately. A tool of an open access medical forum with included VS permits a clearly separated teaching and education of these two different and recharging diagnostic procedures. The selected still images can be compared with the VS (sampling), and the final diagnostic statement with the diagnosis derived from the selected region of interest (ROI) [7,34]. Especially, routine diagnostic work using VS requires a correct (and usually quite slow) sampling process prior to the evaluation of diagnosis [5,6,22]. Additional tools such a multiple choice questionnaires, for example those developed in the digital lung pathology teaching atlas could be included using distinct links [7].

## Conclusions

The time of the released, formerly successful and trend setting medical expert consultation systems such as the AFIP, iPATH, UICC-TPCC seems to be exhausted. Funded by non profit organisations, none of the systems could survive due to financial (AFIP, UICC-TPCC) or internal reasons (iPATH) for more than 10 years. Their replacements or remains seem not take into account the fast development of open access forums designed for flexible individual interactive information exchange. These so - called social forums have developed and are still developing specific expressions in terms of information spread, collection, and its - often unexpected - use. All these features reflect to information exchange in specialized (medical) disciplines too. They can be used to implement comparable forums with specifically designed aims, such as the developed MECES. The perspectives of such systems are promising, especially as they are a useful worldwide accessible tool to communicate in a flexible, save, and familiar manner between different medical disciplines.

## Competing interests

The authors declare that they have no competing interests.

## Authors' contributions

All authors contributed to the development, testis, writing of, and read and approved the final manuscript.
